# Traumatic Penetrating Neck Injury with Right Common Carotid Artery Dissection and Stenosis Effectively Managed with Stenting: A Case Report and Review of the Literature

**DOI:** 10.1155/2018/4602743

**Published:** 2018-06-10

**Authors:** Seidu A. Richard, Chang Wei Zhang, Cong Wu, Wang Ting, Xie Xiaodong

**Affiliations:** ^1^Department of Neurosurgery, West China Hospital, Sichuan University, 37 Guo Xue Xiang Road, Chengdu, Sichuan 610041, China; ^2^Department of Surgery, Volta Regional Hospital, P.O. Box MA-374, Ho, Ghana

## Abstract

**Introduction:**

Penetrating neck injuries (PNI) are common and associated with arterial and other neuronal injuries. Although many authors have written on penetrating and blunt carotid artery injuries as a result of PNI or traumatic neck injuries, no one has reported a case or case series on PNI that resulted in blunt carotid dissection and stenosis.

**Case Presentation:**

We present a case of 40-year-old building and construction male worker who slipped and fell on an iron rod that resulted in penetrating wound on the right side of the anterior neck a week prior to presenting at our facility. He pulled out the iron rod immediately. Computer tomography angiography (CTA) done revealed C2-C4 transverse process fractures on the right side and a fracture at the right lamina of C3 and right common carotid artery dissection with stenosis. He was successfully treated with stenting via endovascular approach.

**Conclusions:**

We adopt the view that patient should never pull out objects that result in PNI because of complex neurovascular architecture of the neck. The mortality rate of our patient will have doubled if the iron rode penetrated the common carotid artery. The gold standard treatment option for carotid artery dissection and stenosis is endovascular approaches.

## 1. Introduction

Penetrating neck injuries (PNI) are common and associated arterial injuries in about 10-25% of cases with the carotid arteries twice as frequent as the vertebral arteries [1–4]. Carotid artery injuries (CAI) constitute approximately 22% of all cervical vascular injuries (CVI) and out of this percentage common carotid artery injuries (CCAI) constitute roughly 75% while internal carotid artery injuries (ICAI) constitute roughly 20% [5–7]. ICAI usually carries death and stroke rates of about 31% and 23%, respectively [5, 7]. The general mortality rate in PNI with associated CVI is about 66%, with mortality and stroke rates frequently seen in ICAI compared with CCAI [5, 6]. CVI are classified into blunt cervical injuries (BCI) and penetrating cervical injuries (PCI) [8–10]. Artery dissection of the carotid arteries occurs when a tear arises in the intima or the media of the arterial wall, resulting in the formation of intramural hematoma in the subintimal, medial, and subadventitial layers leading to narrowing (stenosis) or complete occlusion [11–13]. Computer tomography angiography (CTA) has now proven to be a quick, reliable, and accurate new device used in the assessing of patients with PNI who have associated BCI as well as PCI [14–16]. This device is extremely precise in diagnosing and excluding injuries necessitating intervention. It has also drastically cut down nontherapeutic surgical neck explorations [3, 17–19]. We present case of traumatic PNI with right carotid external artery dissection and stenosis which we effectively managed with stenting.

## 2. Case Report

**Figure 1:**
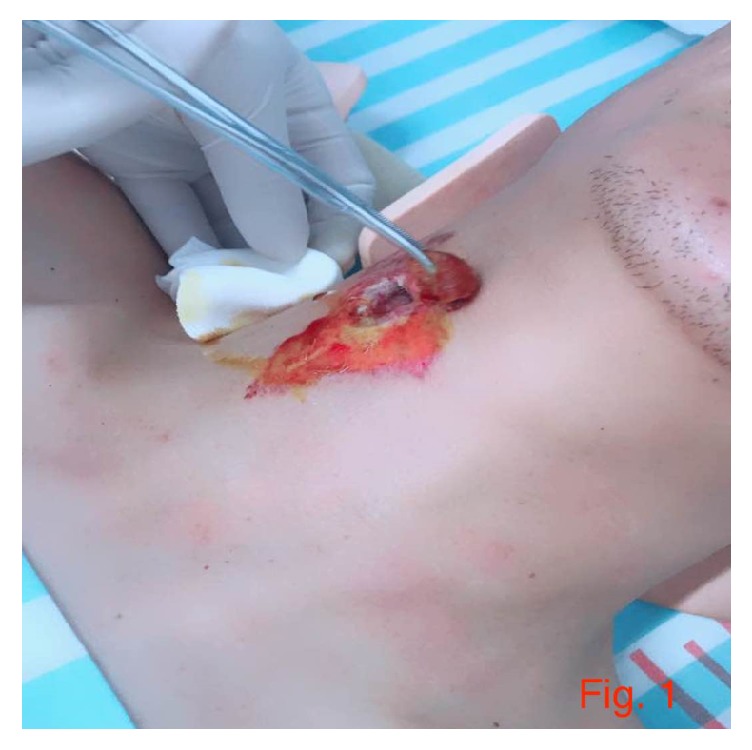
Figure 1 is an image of the wound at the right anterior neck.

We present a case of 40-year-old building and construction male worker who slipped and fell from a height of three (3) meters and sustained a deep penetrating wound on the right side of the anterior neck a week prior to presenting at our facility. He was apparently working from the above height when he slipped and fell on a sharp piece of iron rod which penetrated deep into the right anterior neck. He quickly pulled the sharp iron rod out when he got up from the floor. According to him, the bleeding was not profuse and stopped when he arrived at the local hospital to search for remedy (Figure 1). He did not have hemiplegia, paraplegia, or quadriplegia when we saw him. He is not known to be hypertensive. He did not take alcohol prior to the fall although he takes alcohol occasionally. He had a left femoral fracture at the age of 24 and a right femoral fracture at the age of 32; both incidences were operated on successfully. On examination at our facility we saw a middle aged man who was conscious and alert but however acutely ill with his neck fixed in cervical collar. General as well as systemic examination did not yield much. All the systems where grossly normal. Neurological examination revealed normal pupils which reacted normally to light. Cranial nerves examination was unremarkable. Power on four limbs as well as reflexes was normal. Digital rectal examination revealed a normal spinster tone. Routine laboratory as well as other ancillary (ECG, CXR, etc.) investigations were normal.

**Figure 2:**
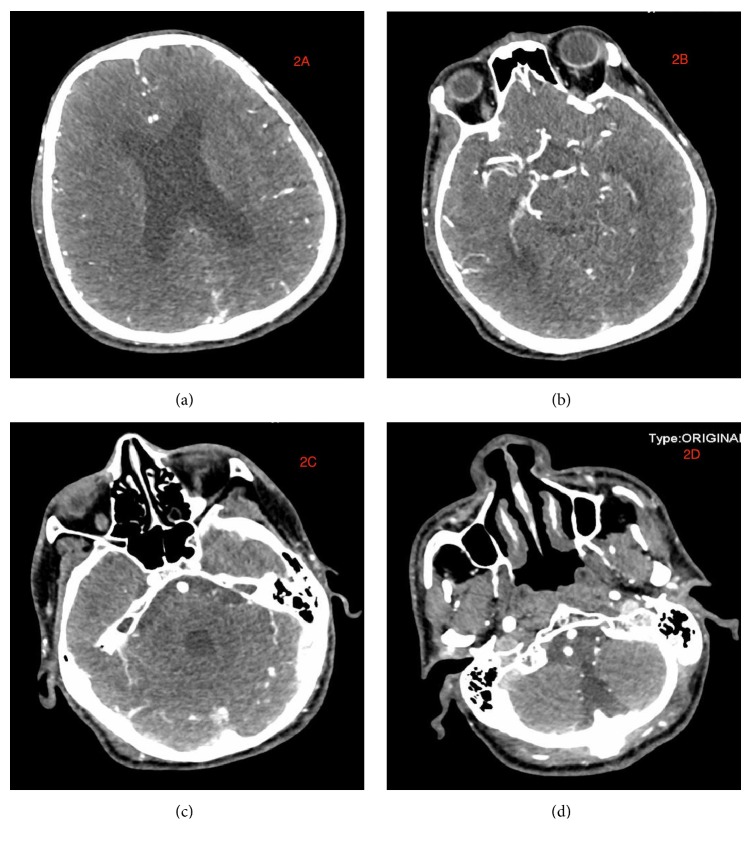
(a)-(d) are CT-scan images indicating no intracranial abnormities after the injury.

**Figure 3:**
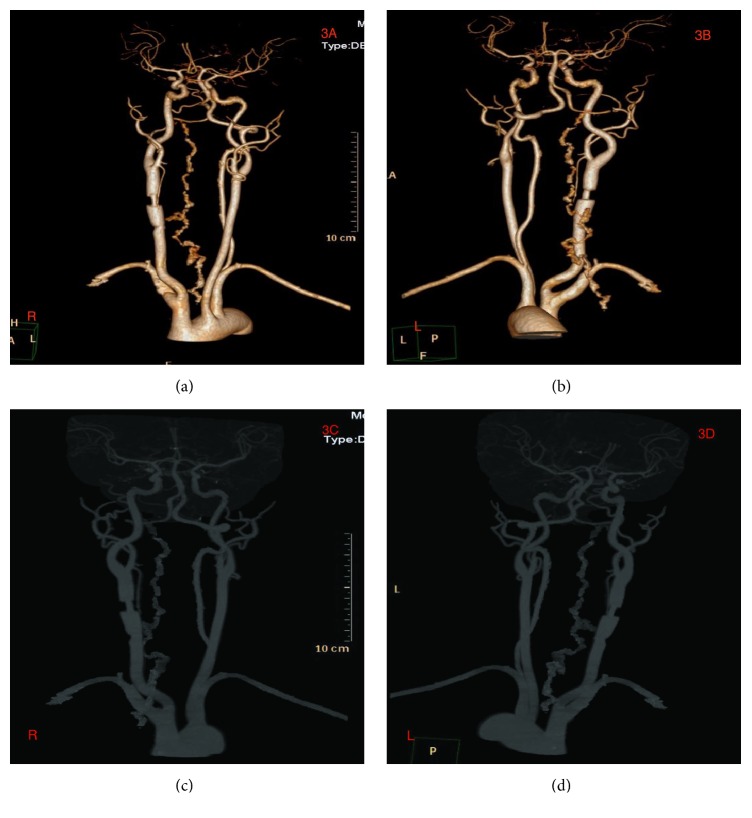
(a)-(d) are DSA images showing right common carotid artery dissection and distorted right vertebral artery.

**Figure 4:**
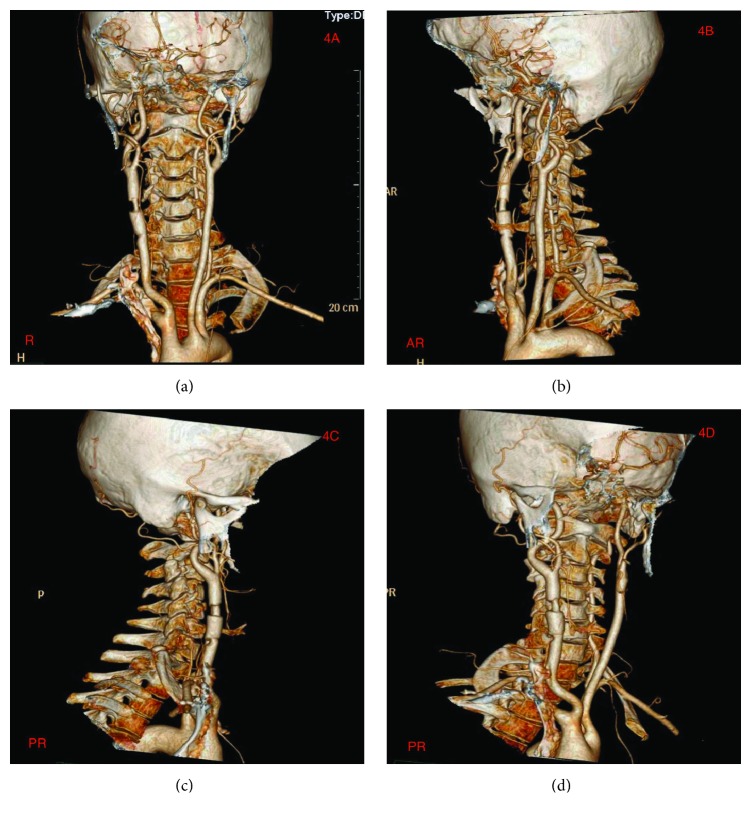
(a)-(d) are CTA showing the fractures at spinous processes of C2-C4 as well as right common carotid artery dissection.

Neck CT-scan done at the local hospital revealed C2-C4 transverse process fractures on the right side, fracture at the right lamina of C3, and right common carotid artery dissection. CT-scan of the head showed no abnormalities (Figures 2(a) and 2(b)). Explorative three-dimensional reconstruction plain and enhanced scan imaging of the cervical spine, chest, and abdomen done at our facility revealed two segmental stenoses of the right common carotid artery with very pale V1 and V3 segment of the right vertebral artery as well as blockage at V2 segment (Figures 3(a)–3(d)) as well as fracture at the right lamina of C3 and C2-C4 transverse processes with free bone fragments and peripheral soft tissue swelling (Figures 4(a)–4(d)). The skin at the right anterior cervical region is discontinuous, with adjacent soft tissue swellings and gas accumulation. The bilateral carotid artery sheath lymph nodes slightly enlarged. At the upper lobe of the right lung there were multiple calcifications, some of which were adjacent to the pleura. There was also slight thickening of the left pleura. The heart was not enlarged but we observed slight accumulation of gas in the anterior mediastinum. Multiple low-density lesions were seen in the liver which we think are constant cysts. A working diagnosis of right common carotid artery dissection with C1-C4 fractures was made.

**Figure 5:**
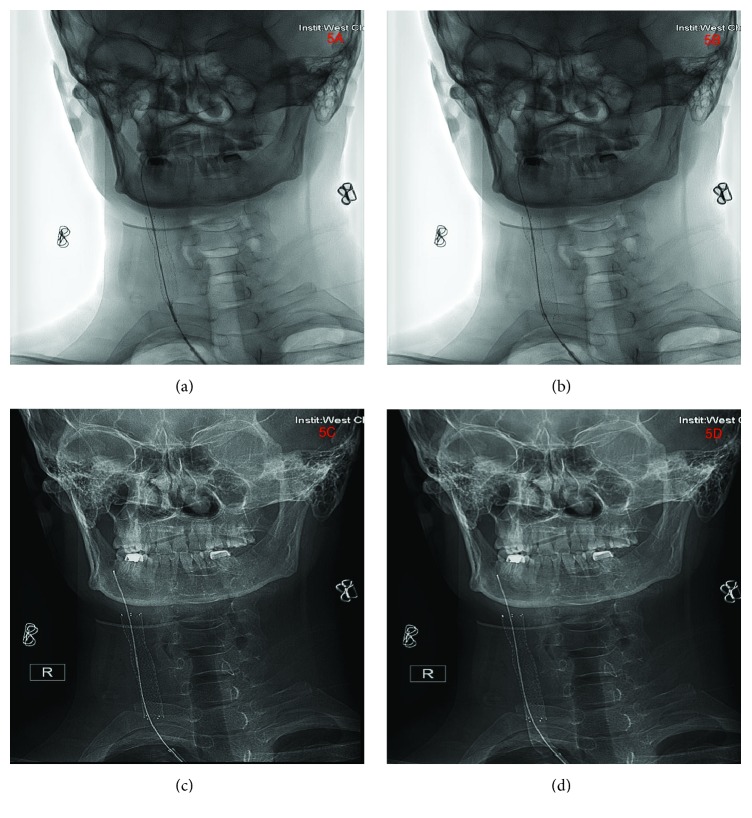
(a)-(d) are postoperative images showing the stent in situ.

**Figure 6:**
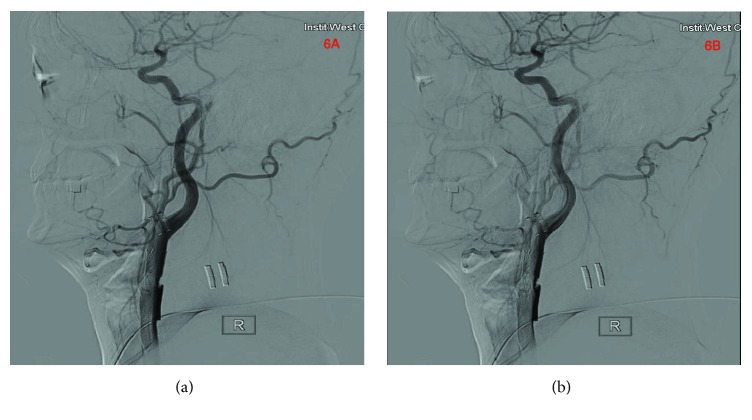
(a) and (b) are control contrasted angiograph done after stenting.

After preoperative education and counselling of the patient as well as the relatives, surgery was scheduled the next day. Intraoperative cerebral angiography showed right carotid artery dissection and right vertebral artery occlusion. There was some reparation at the distal end of the right vertebral artery. The left vertebral artery was however normal. We introduced the guiding catheter guide wire to the proximal end of the right common carotid artery with continued infusion of heparinized saline, after which we introduced a guide wire with a Cordis stent (10 *∗* 60mm) to completely cover the right common carotid artery dissection site with stenosis and released the stent gradually until it completely filled the stenosis area (Figures 5(a)–5(d))). We delivered contrast agent into right common carotid artery to make sure it was patent before removing the guiding catheter followed by withdrawal of the femoral arterial sheath. Control contrasted angiograph done revealed stenting was successful (Figures 6(a) and 6(b)). The patient recovered markedly and was discharged home a week after. Scheduled outpatient visit every 6 months for 2 years revealed no neurological complications.

## 3. Discussion

PNI are common and associated arterial injuries in about 10-25% of cases with the carotid arteries twice as frequent as the vertebral arteries [1–4]. Dissection of the carotid arteries occurs when a tear arises in the intima or the media of the arterial wall, resulting in the formation of intramural hematoma in the subintimal, medial, and subadventitial layers leading to narrowing (stenosis) or complete occlusion [11–13]. CVI are classified into BCI and PCI [8–10]. BCI was originally seen as a very rare traumatic event which occurs in about 0.1% of blunt trauma patients [9, 20]. Blunt CCAI is usually associated with overwhelming neurologic morbidity rate of about 60% and mortality rate of about 19-43% [9, 13, 21]. Penetrating CAI on the other hand occurs in about 4.9-6% of PNI with about 100% mortality rate if left untreated making it extremely fatal [9, 22, 23]. There is usually a decline in mortality rate up to about 6-33% in patients who survive surgery [9, 23]. Our case is puzzling because although it is a PNI, the CCAI is that of blunt jury since the iron rod did not piece it. The optional mechanisms of CCAI are as follows: (i) hyperextension, rotation, or flexion of neck leading to vessel stretch injury; (ii) vessel laceration from bony fracture; and (iii) direct vessel impact [9, 24]. The mechanism of injury in our patient is that of direct vessel impact by the iron rod.

The rise of penetrating neck trauma has led to the conventional division of the neck into three anatomic zones which usually challenged the diagnosis and treatment options [25, 26]: These zones are numbered 1-3. The landmarks in zone 1 start from the clavicles and sternal notch to the cricoid cartilage and house vital structures like the aortic arch, proximal carotid arteries, vertebral arteries, subclavian vessels, innominate vessels, lung apices, esophagus, trachea, brachial plexus, and thoracic duct. The landmarks in zone 2 however start from the cricoid cartilage to the angle of the mandible and house vital structures like the common, internal, and external carotid arteries, the jugular veins, larynx, hypopharynx, and proximal esophagus. Moreover, the landmarks in zone 3 start from the angle of the mandible to base of skull and also house eminent structures like the internal carotid artery, vertebral artery, external carotid artery, jugular veins, prevertebral venous plexus, and facial nerve trunk [3, 8, 9, 14, 25, 27, 28]. It is worth noting that about 80 % of traumatic neck injuries occur at zone 2 while about 10 % occur at zones 1 and 3 [27, 28]. The PNI injury with CCA dissection and stenosis in our case occurred at zone 2.

The carotid artery is accountable for sufficient blood supply to the brain and it is situated adjacent to very crucial neuronal structures in the neck. Bouthillier et al. proposed a segmental classification for the divisions of this artery [9, 29]. To differentiate cervical (C) vertebral arteries from carotid arteries we will use “V” to denote carotid artery. In this classification, V1 denotes the cervical segment of the internal carotid artery (ICA) which commences at the bifurcation of the common carotid artery (CCA). This segment is situated adjacent to cranial nerves IX, X, XI, and XII and the sympathetic chain. The V1 segment of ICA travels deep into the mandible and then enters the skull base medial to the styloid process through the carotid canal after bifurcation from the CCA at the level of hyoid [9, 29]. V2 denotes the petrous segment of the ICA which travels within the petrous portion of the temporal bone until it reaches the foramen lacerum [9, 29]. V3 denotes the lacerum segment of ICA which also commences from the superior part of foramen lacerum and extends to the petrolingual ligament, which is made up of a reflection of the periosteum between the lingula and petrous apex of sphenoid bone [9, 29].

V4 also denotes the cavernous portion of ICA which travels within the cavernous sinus in close approximation to cranial nerves III, IV, V_1_, V_2_, and VI. Furthermore, V4 travels along the lateral and superior side walls of sphenoid sinus in a posterior-to-anterosuperior direction and exits medial to the anterior clinoid process to become intradural segment [9, 29]. V5 denotes the clinoid segment of the ICA which begins at the cavernous sinus and exits at the proximal dura ring. V6 denotes the ophthalmic segment of the ICA which travels superomedially with the optic nerve and bifurcates into the ophthalmic artery and superior hypophyseal artery [9, 29]. V7 denotes the communicating segment of the ICA which rises from the posterior communicating artery and bifurcates into the anterior cerebral artery and the middle cerebral artery. The V7 portion of the ICA also bifurcates into the anterior choroidal artery and the posterior communicating artery [9, 29].

Enough patency and flow through the ICA are fundamental for brain function and survival in patients with traumatic neck injuries. Therefore, Denver group proposed the most widely accepted classification for BCI in the literature [9, 30]. Their classification is as follows: Grade I in which there is luminal irregularity with less than 25% luminal narrowing, Grade II in which there is greater than 25% luminal narrowing, intraluminal thrombus, or raised intimal flap, Grade III which is associated with pseudoaneurysm formation, Grade IV in which luminal occlusion occurs, and Grade V in which there is vessel transection. This scheme also postulates effective prognostic evidence in evaluating the risks of stroke and mortality [9, 30].

At the time of presentation, about 66-73% of patients who sustain BCI may not show any symptoms; however, the emergence of delayed neurologic symptoms may occur from 1 hour to 7 days after trauma. Furthermore, the inception of ischemic incidents may vary from a few minutes to about one month after injury, with about 82% emanating within the first 7 days [9, 13, 31]. Comparatively, in PCI fetal symptoms usually occur immediately and result in 100% mortality. About 33.7% of patients who sustain CCAI may present with an ischemic event such as transient ischemic attack (TIA) or stroke before reaching the hospital [9, 13]. Other manifestations of CCAI may include ipsilateral headache, Horner syndrome, neck pain, bruit, and tinnitus [9, 13, 32–34]. At time of presentation our patient did not show major symptoms apart from neck pain due to the vertebral fractures he sustained.

CTA, magnetic resonance angiography (MRA), conventional angiography, and Doppler ultrasonography are the four most appropriate imaging modalities used in evaluating patients who sustain BCI. Four-vessel DSA is the most preferred imaging modality as compared to the others though most neuroradiologists encourage the use of CTA as the preliminary evaluation modality in patients who sustain BCI [9, 35–37]. DSA does give the physician not only the capabilities of ruling out the existence of BCI but also a very suitable intervention or endovascular treatment option. However, due to the invasiveness and about 1 % complication rate of DSA as well as its scarcity in many hospitals, it is not widely used. Comparatively, due to the easy accessibility of CTA most clinicians prefer using it as first imaging modality followed by DSA [9, 35, 36, 38]. MRA may be the best initial evaluation imaging modality in deprived hospitals that do not have CTA or DSA. It has the superiority of circumventing radiation exposure so it is most optional imaging modality in evaluation of children who sustain BCI [9, 39]. Doppler ultrasonography on the other hand is not a passable preliminary imaging modality because of its low sensitivity [9, 40]. However, it is very advantageous as a follow-up imaging modality.

The targets of management in BCI patients comprise reducing the advancement of vessel injury, reducing the occurrence of ischemic incidents in patients who have no symptoms, and improving overall neurologic and survival outcomes [9]. The Denver group classification is mostly used in the selecting treatment options for patients who sustain BCI. Based on this classification, antiplatelet or anticoagulant agents are generally used in Grade I and II lesions while simultaneous antithrombotic therapy and endovascular intervention are used in Grade III and above. The Western Trauma Association and the Eastern Association of Surgery of Trauma advocate antithrombotic therapy for Grade I and II injuries in case without contraindications to anticoagulation. Watchful waiting is not advocated again [9, 35, 36].

Numerous retrospective case series on endovascular intervention revealed a good outcome in the treatment of traumatic carotid injuries with moderately low hitches [9, 41–45]. Currently, the gold standard treatment option for BCI is endovascular approach with stenting. We also have successfully managed our case with a good outcome and no complication. Open surgical procedure is the gold standard for management of all zones of penetrating carotid injuries and there is currently a rising trend to the combined endovascular and open surgical approach [9, 46, 47]. Although our patients had PNI, the CCAI was that of a blunt type and not penetrative type. Open surgical approaches conventionally encompass surgical repair or surgical ligation. Whenever possible, all efforts must be geared towards surgical repair because it provides a better chance of survival with reduced risks of permanent neurologic deficits. Surgical repair comprises primary arteriorrhaphy, end-to-end anastomosis, vein grafting, polytetrafluoroethylene (PTFE) patching, and transposition of external carotid to injured ICA [9, 23, 48].

During open surgery, the ICA can be an advantageous landmark because it is devoid of arteries in contrast with the CCA with numerous bifurcations. In contrast with ICA, surgical ligation of CCA can be achieved safely without any complications associated with cerebral perfusion [9, 29]. In many cases, ligation or acute occlusion of the ICA is inadequately achieved with lowly collateral blood flow. Surgical ligation is connected to conspicuously advanced rates of mortality and stroke and therefore should only be earmarked for circumstances where surgical repair is not possible. Endovascular procedures have recently been used in managing PCI, either alone or in combination with an open approach, with encouraging outcome. Many clinicians propose the use of endovascular balloon occlusion to assist detecting the site of transection and to temporarily occlude the proximal, injured segment for a more meticulous open exploration [9, 46]. Endovascular procedure is the gold standard in management of zone 3 neck injuries which are usually very close to the skull base making surgical exposure and repair of the distal ICA more problematic. Furthermore, an endovascular method is advantageous because it can be carried out without general anesthesia, approving access to the patient's neurologic status monitoring during surgical intervention in some patients [9].

## 4. Conclusion

Although many authors have written on PCI and BCI as a result of PNI or traumatic neck injuries, no one has reported a case or case series on PNI that resulted in blunt carotid dissection and stenosis. We adopt the view that patient should never pull out objects that result in PNI because of complex neurovascular architecture of the neck. The mortality rate of our patient will have doubled if the iron rode penetrated the CCA. The gold standard treatment option for carotid artery dissection and stenosis is endovascular approaches.

## Abbreviations

PNI: Penetrating neck injuries
CTA: Computer tomography angiography
CAI: Carotid artery injuries
CVI: Cervical vascular injuries
CCAI: Common carotid artery injuries
ICAI: Internal carotid artery injuries
BCI: Blunt cervical injuries
PCI: Penetrating cervical injuries
ICA: Internal carotid artery
CCA: Common carotid artery
TIA: Transient ischemic attack
MRA: Magnetic resonance angiography
PTFE: Polytetrafluoroethylene.
